# Earth and life evolve together—a comment on Yamahira *et al*.

**DOI:** 10.1098/rsbl.2021.0568

**Published:** 2022-03-30

**Authors:** Ralf Britz, Lynne R. Parenti, Lukas Rüber

**Affiliations:** ^1^ Senckenberg Natural History Collections Dresden, Museum of Zoology, Dresden, Germany; ^2^ Division of Fishes, Department of Vertebrate Zoology, National Museum of Natural History, Smithsonian Institution, Washington, DC 20560, USA; ^3^ Naturhistorisches Museum Bern, 3005 Bern, Switzerland; ^4^ Aquatic Ecology and Evolution, Institute of Ecology and Evolution, University of Bern, 3012 Bern, Switzerland

## 1. Introduction

**Figure 1.**
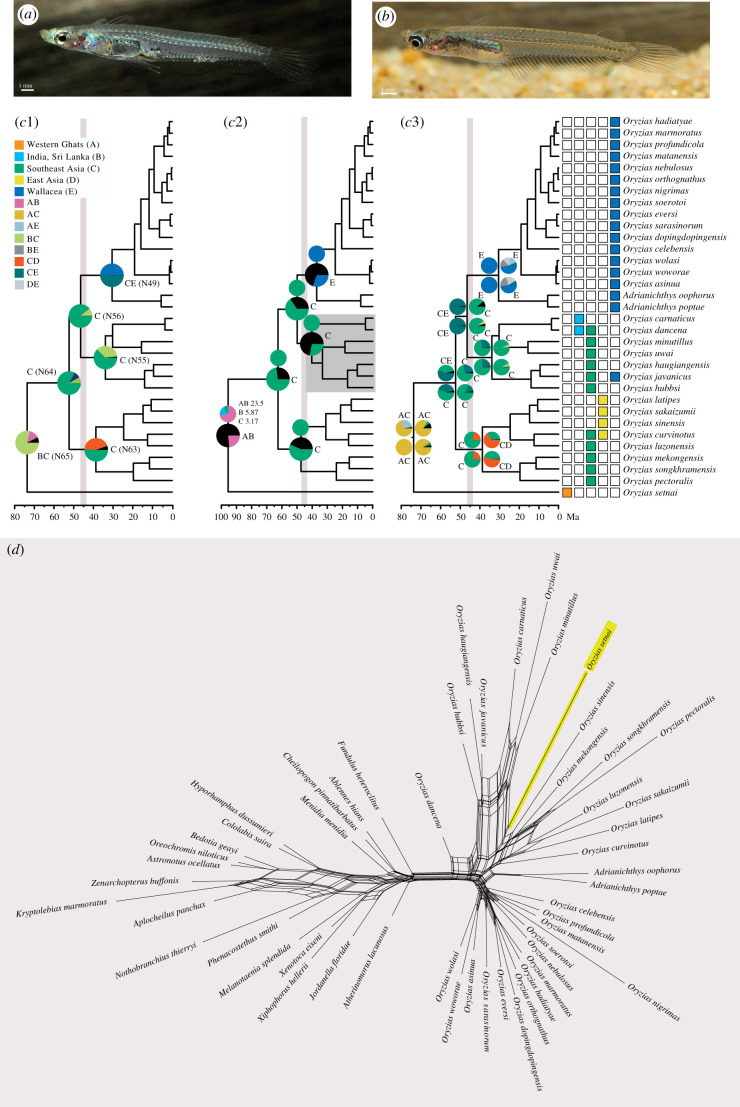
(*a*) Live male and (*b*) live female of *Oryzias setnai*. (*c*) Ancestral area reconstruction shown as pies for selected nodes using RASP. Most likely reconstructions indicated next to pies. Grey lines mark opening of Makassar Strait as cut-off at 45 Mya used for stratified analyses. Note split between *Celebensis* + *Javanicus* groups predates opening of Makassar Strait due to normal distribution prior in their BEAST [4] analysis. (*c*1) RASP analysis (DEC) as described by Yamahira *et al.* [1] (table 1, analysis 1). Selected node numbers (N65, N64, N63, N56, N55, N49) as in table 1. (*c*2) RASP analysis (DEC + J) applying scaling factor 100× to branch lengths (table 1, analysis 2). Smaller pies, omitting black pie areas, correspond to their fig. 21. (*c*3) Four different RASP analyses (all DEC, table 1, analyses 5, 9, 13, 17). (*d*) Neighbour-net using LogDet distances based on their dataset. *Oryzias setnai* highlighted in yellow.

The provocative study by Yamahira *et al*. [1] hypothesizes that one species, *Oryzias setnai* (figure 1*a*,*b*), endemic to coastal areas of west-flowing streams of the Western Ghats, is the sister species of all other ricefishes and that it diverged in the late Mesozoic. They conclude India is the centre of origin of ricefishes, the ancestral lineage of which subsequently diversified and dispersed to occupy its current broad range throughout Asia and Southeast Asia. This scenario is presented as the only possible conclusion from the molecular phylogenetic analysis. We challenge their scenario and conclusions based on a reanalysis of their data.

## 2. Biogeographical reanalysis

Using the information provided in the main article and supplementary file (electronic supplementary material), we were unable to reproduce Yamahira *et al*.'s [1] biogeographical results with their settings and constraints for their dataset as in their fig. 2. Instead, we obtained the ancestral areas illustrated in our figure 1*c*1 (table 1, analysis (1). After contacting the authors about this discrepancy, we received input files that enabled us to reproduce their results (our figure 1*c*2 and table 1, analysis (2)), but the branch lengths of the tree input file were modified and scaled by a factor of 100×, information omitted from their paper. We also noted that the number of decimal points in the branch lengths of this scaled tree input file exceeded six decimal points, a format commonly used. Using branch lengths rounded to six decimal points without or even with a scaling factor of 100× again produced our result (figure 1*c*1 and table 1, analyses 3, 4), not theirs. These inconsistencies strongly suggest that the result of Yamahira *et al*. is an artefact of their RASP [2] analysis due to a combination of unnecessary branch scaling and branch length decimal points. For reason unknown to us, RASP is unable to produce consistent results, although BioGeoBEARS [3] provides consistent results for all datasets (N. Matzke, 2021 personal communication).

**Table 1.** Summary of RASP analyses (1–20) using different parameter settings, range constraints, time stratification strategies, and models (DEC or DEC + J) for selected nodes. The optimal model using modeltesting under the AICc_wt criterion is indicated by underline for each analysis. Ancestral area combinations >10% are listed and most likely states are highlighted in different colours. For analysis 2, the tree from analysis 1 was scaled and for analysis 4, the tree from analysis 3 was scaled.

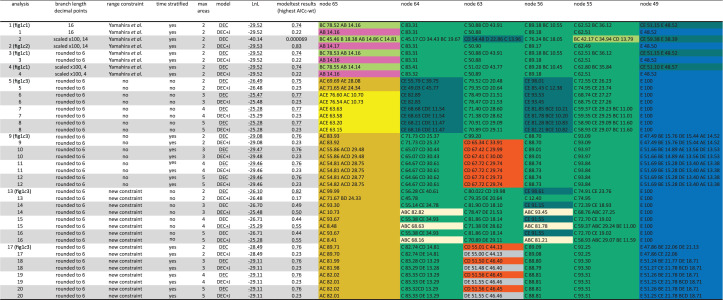

We also note that their pie charts that provide likelihoods of the different ancestral area reconstructions at the nodes in their fig. 2 do not represent the actual output results, but modifications that omit the large number of area reconstructions prohibited by their constraints (shown as black areas in pies in our figure 1*c*2). For example, the pie at node 65 in our figure 1*c*2 (last common ancestor of Adrianichthyidae, their node 2) shows *ca* 70% for area A + B in their fig. 2, but its likelihood is much lower at only 23.51% (figure 1*c*2).

To further explore the influence of range constraints, time stratification and maximum number of areas allowed on the ancestral area reconstruction, we performed 16 additional RASP analyses. The results of four of these are illustrated in figure 1*c*3 (table 1, analyses 5, 9, 13, 17). In none of these 16 additional analyses were we able to obtain the Western Ghats + India (AB) as the ancestral area (table 1). Rather, a variety of ancestral areas were recovered. This means that the RASP analysis of their dataset does not support an out-of-India scenario for Adrianichthyidae, the main result and conclusion of their study and that the ancestral area reconstruction depends heavily on the input parameters.

Notwithstanding these problems with Yamahira *et al*.'s [1] analysis, we take issue with their premise that shared biotic taxa between India and Southeast Asia may be explained only by dispersal either ‘out-of-India' or ‘into-India'. Even with support for *O. setnai* as the sister group of all other ricefishes, these are not the only possible explanations for the distribution pattern. Vicariance—the differentiation of a widespread, ancestral ricefish distribution by geological and climatic processes—is a principal, and here likely, mechanism of biogeography, yet it is ignored. Yamahira *et al*. [1] even chose parameters for their analysis that precluded such a scenario by restricting the number of areas that a species may occupy to two.

A revision of their study is necessary for which we also recommend addressing the following issues:

## 3. Phylogenetic position of *Oryzias setnai*

Yamahira *et al*. [1, p. 2] contend that: ‘Though the endemism of *O. setnai* suggests long-term isolation, no study has investigated its phylogenetic position or evolutionary history.' This is false. Parenti [5] inferred that *O. setnai* is phylogenetically embedded among a group of diminutive ricefishes and in a sister group relationship with *O. uwai* from Myanmar. This [5, p. 538] ‘…represents the first explicit statement of the phylogenetic relationships of *O. setnai* to other ricefishes'.

Yamahira *et al*. confirmed the extreme genetic divergence of this species [1, p. 3]: ‘The branch of *O. setnai* in these [molecular phylogenies] was disproportionately longer compared with other adrianichthyids'. That *O. setnai* was recovered as the sister group of all other ricefishes in a molecular phylogenetic analysis with high branch support, therefore, is not surprising and possibly reflects a long branch attraction artefact [6] (see [7] for a similar example). Support for the phylogenetically uncertain position of *O. setnai* may be gained from our phylogenetic network analysis of their mitochondrial + nuclear dataset [8], in which this species is not opposite all other adrianichthyid species, but rather in its middle.

To explain the exceptionally long branch of *O. setnai*, Yamahira *et al*. [1] invoked a species bottle-neck caused by Deccan Trap vulcanism, an untested hypothesis, not an explanation of evolutionary divergence.

## 4. Calibration

The authors employed three fossil calibrations including †*Lithopoecilus brouweri*, a fossil of Miocene age from Sulawesi described by de Beaufort [9] as intermediate between *Oryzias* and the Sulawesi endemic *Adrianichthys*. Like Rosen [10], Parenti [5] included this fossil in the Adrianichthyidae, but only tentatively. In contrast, Yamahira *et al*. [1] used †*Lithopoecilus* to calibrate the internal node between *Oryzias sarasinorum* and *Oryzias eversi*, citing Horoiwa *et al*. [11]. The latter considered †*Lithopoecilus* to represent the last common ancestor of these two recent species without any supporting evidence. Its use for calibration of this internal node is unfounded.

In conclusion, the ‘out-of-India' dispersal hypothesis to explain modern ricefish biogeography is unsupported and vicariance, the fragmentation of a coastal widely distributed ancestral species by tectonic and climatological events, a better explanation for the historical biogeography of ricefishes.

## Data Availability

Datasets and result files for the analyses in this paper have been deposited on Dryad Digital Repository https://doi.org/10.5061/dryad.v6wwpzgxd [12]. The data are provided in electronic supplementary material [13].
